# The Safety Profile of Sodium-Glucose Cotransporter-2 Inhibitors and Glucagon-like Peptide 1 Receptor Agonists in the Standard of Care Treatment of Type 2 Diabetes Mellitus

**DOI:** 10.3390/life13030839

**Published:** 2023-03-20

**Authors:** Teodor Salmen, Florin-Teodor Bobirca, Ioana-Cristina Bica, Doina-Andrada Mihai, Corina Pop, Anca Pantea Stoian

**Affiliations:** 1 Doctoral School of Carol Davila, University of Medicine and Pharmacy, 050474 Bucharest, Romania; teodor.salmen@drd.umfcd.ro; 2 Department of General Surgery, Carol Davila, University of Medicine and Pharmacy, 050474 Bucharest, Romania; 3 Department of Diabetes, Nutrition and Metabolic Diseases, Carol Davila University of Medicine and Pharmacy, 050474 Bucharest, Romania; anca.stoian@umfcd.ro; 4 Department of Gastroenterology and Internal Medicine, Carol Davila, University of Medicine and Pharmacy, 050474 Bucharest, Romania; cora.pop@umfcd.ro

**Keywords:** antidiabetic drugs, safety, GLP-1 RAs, SGLT-2i, metformin

## Abstract

Aim: We evaluated the safety of sodium-glucose cotransporter-2 inhibitors (SGLT2i) and glucagon-like peptide 1 receptor agonists (GLP-1 RAs) for their use with other glucose-lowering drugs and drugs for the treatment of type 2 diabetes mellitus (T2DM), in a standard-of-care regimen with maximum tolerated doses, and, respectively, when compared with metformin. Methods: We conducted a retrospective, observational study on 405 patients that were seen in the outpatient clinic of the N Paulescu National Institute for Diabetes Mellitus, Bucharest, Romania, in 2019. Their demographics, metabolic parameters, and medication safety were evaluated at three follow-up visits, from baseline, six months, and twelve months. Results: Both SGLT-2is and GLP-1 RAs are safe regarding creatinine, eGFR, urea, GOT, and GPT upon the comparison of the data from the six- and twelve-month visits with the initial visit, and also the twelve-month visit with the six-month visit. Moreover, when comparing SGLT-2is and GLP-1 RAs with metformin, there are safety data only for urea. Conclusions: In this retrospective analysis, both SGLT-2is and GLP-1 RAs, when used in conjunction with other glucose-lowering, blood-pressure-lowering, and lipid-lowering medications, appeared to be safe for the management of T2DM.

## 1. Introduction

Chronic, non-transmittable disorders, such as type 2 diabetes mellitus (T2DM), obesity, heart disease, stroke, cancer, chronic respiratory diseases, and mental health disorders, are epidemics in modern society [1]. T2DM is a part of the cardiovascular (CV), renal, and metabolic diseases, and it is characterized by numerous comorbidities; alongside its continuously increasing incidence worldwide, it requires multiple classes of drugs for its treatment, which is a considerable deterrent to polypharmacy [2]. Polypharmacy is a term that is used to define the prescription of more drugs at the same time. Usually, it is referred to as the administration of at least five drugs simultaneously [3,4].

Polypharmacy favors adverse and drug–drug interactions, reduces reported adherence and compliance, and has a severe impact, because it can lead from a simple fall to fractures, an increased need for hospitalization, drug toxicity, renal or liver insufficiency, heart failure, cognitive decline, and even death. In addition, patients with T2DM are frail due to the disease’s long and asymptomatic evolution and its multiple comorbidities. Therefore, its case management should not aggravate the underlying diseases, which can be achieved through a personalized and safe pharmacotherapy solution [5,6,7,8,9,10,11,12,13].

In T2DM, the pathophysiological course of the disease includes a progressive decrease in insulin secretion. Secondary to the remaining endogenous secretion of insulin, the tendency is to prioritize treatments with exogenous insulin. The cases of acute conditions, either of medical or surgical origin, that are associated with an acute glycemic disequilibrium, require mandatory endogenous insulin treatment. Otherwise, the tendency is to prolong the benefits of non-insulin therapies as much as possible. This approach is encouraged by the latest development in antidiabetic non-insulin drugs that have had proven benefits for weight, blood pressure (BP), metabolic equilibrium, and lipids metabolism, alongside other pleiotropic effects, which constitute the primary alternatives of the standard-of-care treatment for T2DM [14]. Moreover, the drug–drug interactions between BP-lowering medications and non-insulin antidiabetic drugs are clinically non-significant for valsartan [15], ramipril [16], verapamil [16], or statins [15] in healthy participants. However, more data are needed for statins to eliminate potential interactions such as myopathy [17].

The two classes of novel antidiabetic non-insulin drugs that are important for the standard-of-care treatment of T2DM are sodium-glucose loop transporter 2 inhibitors (SGLT-2i) and glucagon-like peptide 1 receptor agonists (GLP-1 RA). Their development is secondary to CV outcome trials (CVOT), which, despite their proven non-inferiority effects in terms of glycemic and metabolic control, have shown that they also offer CV protection and that they decrease the need for hospitalization, which are benefits of the utmost importance for patients with T2DM [18,19,20,21,22,23]. These are in contrast to the old treatments for T2DM that are reported to increase the risk of hypoglycemia, hospital admission, and death associated with hospitalization, especially in old and frail patients [24].

Even though the reported data from the literature are favorable for these two new classes, data on their safety profiles are scarce, as these are frequently evaluated as secondary outcomes. SGLT-2is are reported to reduce 3-point major adverse cardiovascular events (3-point MACE), the risk of CV and all-cause mortality, and hospitalization for heart failure in several large, randomized control trials (RCT), respectively, with EMPA-REG OUTCOME for empagliflozin, DAPA-HF and DAPA-CKD for dapagliflozin, and CREDENCE and CANVAS for canagliflozin [25,26,27]. Simultaneously, the safety parameters demonstrated for SGLT-2i, in relation to renal function, are the decreasing of the incidence of worsening nephropathy, the doubling of serum creatinine, and the need for renal-replacement therapy for empagliflozin in EMPA-REG OUTCOME. Moreover, a slower eGFR rate decline for dapagliflozin in DAPA-HF; a reduction of albuminuria and of the risk of kidney function loss, and a slower decline of the eGFR for canagliflozin in CANVAS are reported as safety parameters. In relation to hepatic function, there is a reported reduction of aminotransferases (GPT > GOT), which are secondary to hepatic fat reduction for empagliflozin in EMPA-REG OUTCOME, and of GOT, GPT, and γ-glutamyl transpeptidase (GGT) for canagliflozin in CANVAS, alongside with classical adverse reactions (AR) such as reducing the incidence of hypoglycemia episodes, volume depletion, arrythmias, amputation, urinary tract infections, and all-cause mortality [20,26,27,28,29,30]. On the other hand, GLP-1 RAs are reported to reduce 3-point MACE and the rate of myocardial infarction and non-fatal stroke for semaglutide in PIONNER and SUSTAIN, and for liraglutide in LEADER [31,32,33]. In terms of safety, there are data regarding the low incidence of renal function alteration for semaglutide in SUSTAIN and liraglutide in LEADER, but no recorded significant changes on eGFR and for a decrease in UACR for semaglutide in PIONEER. There are also data on the reduction in microalbuminuria for semaglutide in LEADER and REWIND, and for liraglutide in LEADER, and on the lowering of the rate of doubling and persistent doubling of creatinine levels, or the progression towards end-stage renal disease and death from the renal disease for liraglutide in LEADER, and on safety in the case of administration for renal insufficiency for dulaglutide in AWARD. In relation to hepatic function, there is no need for a dose adjustment in the case of hepatic insufficiency for semaglutide in SUSTAIN and PIONEER, and dulaglutide in AWARD, there is a reduction in liver fat content, improvement of GGT level, and non-significantly reduction of GOT, GPT, and liver stiffness for semaglutide in D-LIFT, with safety for retinopathy, pancreatitis, hypoglycemia events, and gastrointestinal AR such as nausea, vomiting, or bloating [31,32,33,34,35,36,37].

This study aimed to evaluate the safety of two glucose-lowering medications, namely, SGLT-2is and GLP-1 RAs, which are administered for treating patients with T2DM in a real-world setting, according to the standard-of-care regimen with maximum tolerated doses, in association with other antidiabetic, BP-lowering, and lipid-lowering medications.

## 2. Materials and Methods

This retrospective, observational study was conducted following the Declaration of Helsinki and approved by the Institutional Ethics Committee of N Paulescu National Institute for Diabetes Mellitus, Nutrition and Metabolic Disorders, Bucharest, Romania (protocol number 5591, from 17 November 2022). It included 405 patients with T2DM that were treated with standard-of-care treatment with the maximum tolerated doses, consecutively admitted during 2019 to the N Paulescu National Institute for Diabetes Mellitus, Nutrition and Metabolic Disorders’ Outpatient Department. The patients were included if they met the inclusion and exclusion criteria. The inclusion criteria were represented by adult patients with an established T2DM diagnostic, at least six months prior to admission, who had been treated with the standard-of-care treatment for at least 6 months before the baseline visit, and had undergone at least two of the three visits of interest and who received at least one of the BP-lowering or lipid-lowering drugs of interest. These are extensively presented in Table 1. The exclusion criteria were mainly centered around the patients with other types of DM who were not adults, as shown in Table 1. The interest drugs from the SGLT-2i and GLP-1 RA classes are the ones that were available and approved by the National Drug Association, respectively, empagliflozin and dapagliflozin for SGLT-2i, and dulaglutide, lixisenatide, and exenatide for GLP-1 RA.

The patients’ data were collected from the hospital’s electronic database. The parameters of interest included demographic elements (e.g., age, gender, and settlement), clinical data (palpitations, presence of hypoglycaemia, and gastrointestinal AR, such as nausea, diarrhea, vomiting, or the appearance of pancreatitis or urinary tract infections), comorbidities (e.g., high BP (HBP) and dyslipidemia, etc.), paraclinical elements (creatinine, estimated glomerular filtration rate (eGFR), urea, transaminases (GOT–aspartate amino-transferase, GPT–alanine amino-transferase), and urinary albumin to creatinine ration (UACR)), and data about the treatment (antidiabetic, BP-lowering, and lipid-lowering drugs). These were observed, respectively, at baseline, six-month, and twelve-month visits. Furthermore, the data were systematized as Excel tables and analyzed using both Excel and PSPP software.

## 3. Results

The study group included 405 patients, and data about their distribution, demography, and the CV treatment of their interest distribution characteristics are summarized in Table 2.

**Figure 1:**
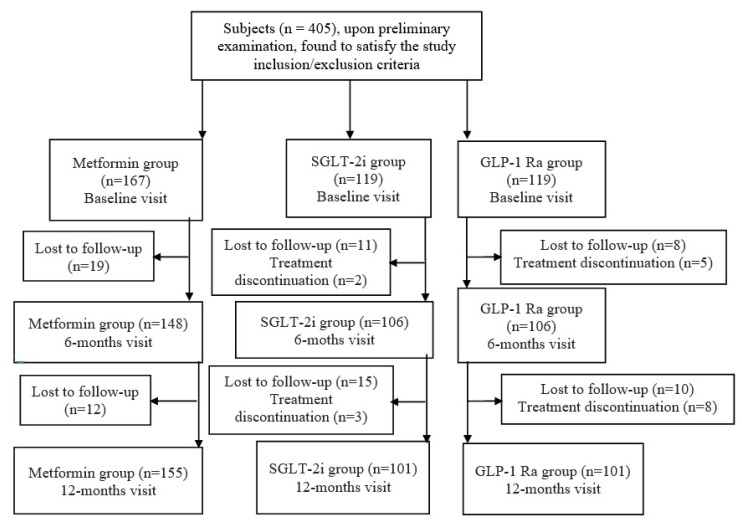
Intention to treat of the analyzed patients. SGLT-2i—sodium glucose loop transporter 2 inhibitor; GLP-1 RA—glucagon-like peptide 1 receptor agonist.

The analysis of the patients’ intention to treat is synthetized in Figure 1, which presents, respectively, the included patients, those lost after follow-ups, and those with discontinued treatment due to AR.

### 3.1. Metformin, SGLT-2i and GLP-1 RA

The baseline characteristics of safety included a few ARs such as palpitations (two patients in the SGLT-2i and GLP-1 RA groups, respectively), hypoglycemia (one patient in the SGLT-2i and GLP-1 RA groups, respectively), and those without gastrointestinal ARs or urinary tract infections, while the biologic parameters that were evaluated for the safety assessment were, respectively, creatinine, eGFR, UACR, urea, GOT, and GPT, and are also summarized in Table 3.

The 6-month characteristics of safety included a few ARs, such as hypoglycemia (one patient in the SGLT-2i and GLP-1 RA groups, respectively), with five cases of nausea in the GLP-1 RA group, two cases of urinary tract infections in the SGLT-2i group, and those without palpitations, alongside the biologic parameters of safety, which were, respectively, creatinine, eGFR, UACR, urea, GOT, and GPT, as seen in Table 4.

The 12-month characteristics of safety lacked hypoglycemia and palpitations, but included a few gastrointestinal ARs, which were, respectively, five episodes of nausea in the GLP-1 RA group and two cases of urinary tract infections in the SGLT-2i group, as seen in Table 5. The biologic parameters that were evaluated for the safety assessment, were, respectively, creatinine, eGFR, UACR, urea, GOT, and GPT. Those parameters from the V12M are synthetized in Table 5.

The patients’ safety data from the 6-month visits (V6M) and 12-month visits (V12M) were compared to the safety data from the baseline visits, and also, the safety data from V12M were compared to the safety data from V6M, and the results are shown in Table 6.

For the AR, respectively, although there were no significant differences for palpitations and the presence of hypoglycemia, there were for a few gastrointestinal ARs. In the GLP-1 RA group, there were five cases of nausea at V6M and eight cases of nausea at V12M, while in the SGLT-2i group, there were two cases of urinary tract infections at V6M and three cases of urinary tract infections at V12M, as seen in Table 4 and Table 5.

The renal safety was evaluated using creatinine, and showed significant values for the metformin at V6M and V12M, as compared to the baseline visit (V0M). With eGFR, significant values for the metformin were shown at V6M and V12M, as compared to V0M. For urea, there were significant values for the metformin at V12M, as compared to V0M, and at V12M, as compared to V6M. For SGLT-2i at V6M, there were significant values as compared to V0M, and for GLP-1 RA, these were at V6M as compared to V0M. The use of UACR had no significant values, as seen in Table 6.

The hepatic safety was evaluated using GOT and GPT, with significant values for the metformin at V6M as compared to V0M, as seen in Table 6.

### 3.2. SGLT-2i and GLP-1 RA Groups Compared with Metformin 

Furthermore, the patients’ safety data at V6M and V12M, as compared to the baseline parameters, and the safety parameters from V12M, as compared to V6M, were analyzed, as seen in Table 7. The renal (creatinine, eGFR, and UACR) and hepatic (GOT and GPT) safety lacked statistical significance, except when comparing both the SGLT-2i and GLP-1 RA classes with the metformin for urea, *p* = 0.02.

To sum up, SGLT-2is and GLP-1 RAs, along with metformin, are safe for renal and hepatic profiles, when administered to patients with T2DM who receive the standard-of-care treatment with the maximum tolerated doses of BP-lowering and lipid-lowering medication, alongside a few adverse reactions that are specific to each of the classes. In our study, significant results were obtained for renal safety in the cases of creatinine and eGFR, when comparing the metformin with SGLT-2is and GLP-1 RAs at the 6-month and 12-month visits, as compared to the baseline. This was also the case for urea when comparing SGLT-2i at 6 months, as compared to the baseline, and when comparing the metformin at 12 months, as compared to the 6-month and baseline visits. Furthermore, this was seen for the hepatic safety, for both GOT and GPT, when comparing the 6-month visit with the baseline.

## 4. Discussion

Our real-life study evaluated some of the most prescribed antidiabetic drugs, as they were, until recently, recommended by the standard-of-care of the American Diabetes Association (ADA), namely metformin, alongside two novel antidiabetic, non-insulinic drugs, SGLT-2is and GLP-1 RAs, respectively, which have become the first-line recommended antidiabetic drugs by the standard-of-care of the ADA [38].

Most of the literature’s reported data on these three antidiabetic classes are about their efficacy in metabolic control, respectively, in glycemic, glycosylated A1c, or weight amelioration. At the same time, their safety elements need to be evaluated.

Metformin is a biguanide with pleiotropic effects that inhibits hepatic gluconeogenesis, sensitizes insulin action, and acts in the intestinal tract. It is used in patients with T2DM for glycemic control, and, respectively, does not increase their CV, hypoglycemia, or weight gain risks. However, a lactic acidosis increased risk is reported in a subclinical manner, so it is counter-indicated in patients with severe heart, renal, or hepatic insufficiencies. For its ARs, it is important to emphasize gastrointestinal ones such as bloating or diarrhea [39].

SGLT-2i is a class that lowers the glycemic levels in patients with T2DM by blocking glucose renal reabsorption and reducing body weight and BP, without pharmacokinetic ARs [15]. The most frequent ARs in patients with T2DM that are treated with SGLT-2is are volume depletion, euglycemic diabetic ketoacidosis, urinary and genital tract infections, acute kidney injury, and lower-limb amputations [21,40]. CVOTs report that, when administered to patients with T2DM, SGLT-2is reduce their composite outcome, including end-stage chronic kidney disease (CKD), a doubling of the creatinine level, or death from renal or CV causes [18,19]. As compared to patients treated with other classes of antidiabetic drugs, they reduce the risks of all-cause mortality and arrhythmias [20]. In the case of severely ill patients, there are more frequently encountered instances of dehydration, ketoacidosis, or acute kidney injury [21]. SGLT-2is are reported to have pleiotropic effects on the heart, such as delaying the time-dependent decline in systolic function and the development of cardiomyocytes stiffening [19]. Additionally, they have proved to have CV benefits, by improving CV outcomes and decreasing CV, all-cause mortality, 3-point MACE, and the rate of hospitalization for heart failure [25,26,27].

GLP-1 RA is a novel class of non-insulinic antidiabetic drugs that are part of the incretins and have benefits in terms of metabolic, body weight, lipid, and BP control, alongside other pleiotropic effects. From the CV point of view, they are beneficial by reducing 3-point MACE, CV mortality, the rate of myocardial infarction, and non-fatal stroke [31,32,33]. Their pathophysiological mechanisms include an improvement in the balance between insulin and glucagon, and the stimulation of the first hormone and the inhibition of the latter, alongside the inhibition of appetite and the endogenous secretion of glucose [23]. Their other mechanisms, secondary to weight loss, are the amelioration of the pancreatic beta-cells’ inflammatory levels and insulin sensitivity [23]. For example, on in vitro models, GLP-1 RAs have proved to offer neuronal protection and to improve cognitive function [41]. The gastrointestinal ARs represent their major setback as a class and, are, respectively, nausea, vomiting, and diarrhea, which are the most cited, alongside the risk of developing pancreatitis. The data reported for GLP-1RAs in the treatment of T2DM show metabolic, weight, and CV benefits, alongside renal benefits such as a reduction in eGFR and complex renal outcomes [22,23].

Metformin has a low risk of hypoglycemia, but is also reduced to a minimum for SGLT-2is [42,43] and GLP-1 Ras [44,45].

Regarding the transaminase levels, secondary to the SGLT-2i treatment of patients with T2DM, their amelioration is the last part of the process that starts with a reduction in body weight, a decrease in liver fat content with a secondary amelioration of steatosis, and a reduction in GOT and GPT levels [46].

Dutour et al. [47], on 44 patients with T2DM and obesity, followed them for 26 weeks and reported that exenatide ameliorated hepatic function by reducing the hepatic triglycerides content, which appeared to be secondary to the reduction in body weight (r = 0.47, *p* = 0.03). Armstrong et al. [48], on 52 overweight patients with nonalcoholic steatohepatitis (NASH), after 48 weeks of liraglutide treatment, presented the resolution of the histological signs of NASH to be safe and have a good tolerability, and, accordingly, in the Liraglutide Efficacy and Action in NASH (LEAN) trial, its indication should be extended, in order to be used to treat NAFLD. Flint et al. [49] evaluated, at 24, 48, and 72 weeks, 67 patients with T2DM and obesity, who received semaglutide versus a placebo. They observed that semaglutide reduced the liver fat content compared to the placebo (*p* < 0.0001), without ameliorating liver stiffness but with the amelioration of ALT and high-sensitivity C-reactive protein levels. In a randomized control trial on 64 patients with T2DM and NAFLD that received dulaglutide or the standard treatment, Kuchay et al. [50] demonstrated that dulaglutide reduced GGT levels (−13.1 U/L, *p* = 0.025), with no significant differences on ALT (−13.1 U/L, *p* = 0.10), AST (−9.3 U/L, *p* = 0.075) or liver stiffness measurements. Moreover, in patients with NAFLD, dulaglutide provided a reduction in the liver fat content (−32.1% vs. −5.7%, respectively; mean difference −26.4% (95% CI −44.2, −8.6); *p* = 0.004) as compared to the controls.

### 4.1. Creatinine and eGFR

Metformin is a safe drug for treating patients with T2DM, and has been reported with a risk of developing lactic acidosis, but also cited as slowing the deterioration of renal function [51]. In a study by Hsu et al. [52] on 616 patients with T2DM and moderate CKD, who were treated with metformin for at least six months, 132 interrupted their treatment and were considered to be the interruption group, while 484 maintained the metformin and were considered to be the continuation group. It was reported that the metformin favored the increment of creatinine and the decline of eGFR levels; while in our study, the metformin was safe in terms of creatinine at V6M as compared to V0M, at V12M as compared to V0M, for eGFR at V6M as compared to V0M, and at V12M as compared to V0M.

In a study by Carmena et al. [53], SGLT-2i proved to have renal benefits, with a 45% (*p* < 0.0001) reduction in the composite of the worsening of renal function, end-stage renal disease, or renal death, a result that was similar in the group of CV disease and multiple CV risk factors, respectively. A similar reduction of 30% (*p* < 0.00001) was encountered in CREDENCE for the endpoint, which contained the doubling of the serum creatinine level, or renal or CV death for the canagliflozin, as compared to the placebo [53]. For GLP-1 RA, liraglutide, as compared to placebo, in LEADER, reduced the macroalbuminuria incidence by 26%, with a hazard ratio of 0.74, a 95% confidence interval (CI) of 0.6–0.91. Semaglutide, in SUSTAIN-6, decreased the occurrence or aggravation of nephropathy by 36% (*p* < 0.005) in the study group after 2 years of follow-up, and lixisenatide, in ELIXA, significantly decreased albuminuria [53].

### 4.2. Urea

Zhang et al. [54], in a study on 88 patients with T2DM that had diabetic nephropathy and were randomized in a 1:1 manner to either metformin (experimental group) or liraglutide (control group), reported similar values of urea and creatinine at baseline. They assessed that both the parameters had lower values, with a more significant effect in the experimental than the control group (urea (51.83 mg/dL ± 12.43) and creatinine (0.82 ± 0.19) versus urea (73.63 mg/dL ± 17.59) and creatinine (1.01 mg/dL ± 0.26)) (*p* < 0.0001), similar to the ones from our study, where the patients with T2DM that were treated with the metformin had an improved urea level at V12M, as compared to V0M, and at V12M as compared to V6M.

In a review by Rowlands et al. [55], GLP-1 Ras are reported to improve kidney function by an increment in renal blood flow and a secondary amelioration of eGFR, with the prevention of plasma creatinine rise, which is similar to our study results, which show that, in the patients with T2DM that were treated with GLP-1 RAs, their urea levels improved at V6M as compared to V0M.

For SGLT-2is, the review of Nespoux et al. [56] reported that SGLT-2is improve kidney function by lowering body weight, BP, and volume overload, alongside reducing albuminuria, with the long-term preservation of eGFR, and a slight increase in urea levels. Furthermore, our study showed that SGLT-2is in patients with T2DM ameliorates the urea levels at V6M compared to V0M.

### 4.3. GOT and GPT

Feng et al. [57], in a prospective trial on 85 patients with T2DM that were randomized in a 1:1:1 ratio to metformin, liraglutide, and gliclazide, and followed for 24 weeks, reported that the patients treated with the metformin encountered a reduction in baseline GPT (51.01 ± 5.87 UI/L), as compared to the 24-month visit (28.44 ± 3.24 UI/L), *p* < 0.001. In addition, this was seen for the baseline GOT (34.09 ± 3.13 UI/L), as compared to the 24-month visit (22.64 ± 1.64 UI/L), *p* < 0.001. These are similar to our study results, especially for GPT at V6M, as compared to V0M, and for GOT at V6M, as compared to V0M. Moreover, in a meta-analysis of 615 patients by Ghosal et al. [58], 297 received GLP-1 Ras and 318 received the standard-of-care or other active controls. GLP-1 Ra proved to ameliorate GPT (standardized mean difference (SDM), −0.56, 95% CI −0.88 to −0.25, *p* < 0.01) and GOT (SDM, −0.44, SE, 95% CI −0.64 to −0.24, *p* < 0.01), and reduced the liver fat content (SDM, −0.43, 95% CI −0.74 to −0.12, *p* < 0.01) in patients with T2DM and nonalcoholic fatty liver disease (NAFLD). Furthermore, in a real-life study on 283 Korean patients with T2DM and NAFLD, 188 controls and 95 patients were treated with SGLT-2is. Euh et al. [46] reported that SGLT-2is decreased the AST at 9 months (−8 (−14, −2) vs. −4 (−7, 0) international units/L in control group) and ALT at 9 months (−15 (−22, −7) vs. −5 (−9, 0) international units/L in the control group).

### 4.4. SGLT-2i Compared to Metformin

SGLT-2i is reported to be safe, even compared to other antidiabetic drugs. A systematic review and meta-analysis by Milder et al. [59], which included 3749 patients with T2DM who received either SGLT-2i and metformin or metformin alone, reported no differences for the adverse effects (AE), such as drug-related AEs, hypoglycemia, diarrhea, or urinary tract infections. Moreover, in a meta-analysis by Jingfan et al. [60], on 2509 patients with T2DM who received either SGLT-2i and metformin or metformin alone, no significant differences between the two groups were reported in terms of ARs, such as hypoglycemia, urinary tract infections, or hypotension. However, the group of SGLT-2i and metformin presented a higher risk for gastrointestinal ARs than the metformin group.

### 4.5. GLP-1 RAs Compared to Metformin

GLP-1 RAs are reported to have a similar AR to metformin, especially when talking about the gastrointestinal system [61].

Moreover, a meta-analysis by Huthmacher et al. [62], which compared patients with T2DM that were treated with short- or long-acting GLP-1 RAs and insulin versus only insulin, reported a higher percentage of ARs, such as nausea, vomiting, diarrhea, and hypoglycemia in the GLP-1 RA and insulin groups. It is important to emphasize that between the groups treated with the GLP-1 RAs, the short-acting forms presented higher proportions of ARs, such as nausea, vomiting, and hypoglycemia, than the long-acting forms of GLP-1 RA [62].

A systematic review and meta-analysis by Patoulias et al. [63] on patients with T2DM compared SGLT-2i to GLP-1 RA treatment. They reported that the latter had a significantly greater risk of ARs, such as hypoglycemia, nausea, and diarrhea, but with a similar risk for severe hypoglycemia. Moreover, a lower risk for genital infections, primarily mycotic ones, and a lower risk for treatment discontinuation, was reported for GLP-1 RA compared to SGLT-2i treatment [63].

The large multicenter RCT has shown that both SGLT-2i and GLP-1 RA are safe. For SGLT-2i, there are parameters such as hypoglycemia events, which have been seen in DAPA-HF for dapagliflzon [26], in EMPA-REG OUTCOME for empagliflozin and in CANVAS for canagliflozin [18,26,64]. For urinary tract infections, these have been seen in EMAP-REG OUTCOME for empagliflozin, in CANVAS for canagliflozin, and in DAPA-HF and DECLARE-TIMI for dapagliflozin [28,65]. For fractures, they have been observed in DAPA-HF for dapagliflozin and in CANVAS for canagliflozin [26,66]. For volume depletion, this has been recorded in DAPA-HF for dapagliflozin, in EMBLEM for empagliflozin, and in CANVAS for canagliflozin [26,67,68]. For amputation, this has been seen in DAPA-HF for dapagliflozin [26]. For acute kidney injury for SGLT-2is, this has been noticed in EMPA-REG OUTCOME for empagliflozin [69]. For GLP-1 RAs there are parameters such as hypoglycemia events that have been witnessed in PIONNER for semaglutide, in AWARD for dulaglutide, and in LEADER for liraglutide [31,70,71]. For acute kidney injury, this has been seen in PIONNER for semaglutide, in LEADER for liraglutide, and in AWARD for dulaglutide [31,34,70]. For retinopathy, this has been shown in PIONNER for semaglutide [31], and for pancreatitis, in PIONNER for semaglutide, in AWARD for dulaglutide, and in LEADER for liraglutide [31,72,73]. Gastrointestinal AEs, such as nausea, diarrhea, and vomiting, are higher in some studies [62], while in PIONNER for semaglutide, they have a similar incidence with placebo groups, or are majority mild forms, as in STEP for semaglutide [31,74].

Our real-world results confirm that these antidiabetic non-insulin drugs are safe for the treatment of T2DM in a standard-of-care regimen with a maximum tolerated doses, even when administered in association with the CV drugs used for the treatment of HBP, such as BB or CCB, and with ACEI or ARB, or for the treatment of dyslipidemia with statins, or when compared to metformin. Regarding GLP-1 RA patients, these were uncontrolled patients that required a surprisingly high number of insulin treatments. Moreover, it was observed that the insulin need decreased in these patients treated with GLP-1 RA, alongside the ones treated with SLGT-2i.

The observed side effects from our study population are in a small proportion, despite the fact that the included patients were not selected, but were consecutively enrolled, so an explanation can be the small sample of patients. However, in the large RCT, there were similar rates, and, moreover, the small proportion of females from the SGLT-2i group could be linked to a lower sex-specific prescription of these drugs due to a urogenital infections risk.

This study’s strength is its provision of data about the safety profiles of SGLT-2i, GLP-1 Ras, and metformin, which are rarely assessed. At the same time, its limitations are the low number of included patients, alongside the short follow-up period, which included only three visits during one year.

## 5. Conclusions

Alongside metformin, the new antidiabetic non-insulin drugs, namely, SGLT-2is and GLP-1 RAs, are safe for the treatment of patients with T2DM in a standard-of-care regimen with maximum tolerated doses, even if they are combined with other drugs that are used for the treatment of HBP, respectively, BB, CCB, ACEI, or ARB, or with drugs for the treatment of dyslipidemia, such asstatins, or when administered simultaneously with other antidiabetic drugs such as metformin. This fact eases our management of the complex cases of patients with T2DM, which are frequently associated with CV disease, and benefit the most from the effect of these drugs on metabolic, BP, and lipidic equilibrium, and their pleiotropic effects.

## Figures and Tables

**Table 1:** Inclusion and exclusion criteria.

Inclusion Criteria	Exclusion Criteria
Adults over 18 years old	Younger than 18 years old
Positive diagnostic of T2DM older than six months	Type 1 DM or secondary DM
Standard-of-care treatment for T2DM with maximum tolerated doses	Patients that received other drugs with similar adverse effects with SGLT-2i or GLP-1 RA
Minimum 6 months treatment with SGLT-2i and GLP-1 RA prior to the baseline visit	
≥2 visits of the baseline, 6-month and 12-month visits	
Treatment with BB and/or CCB and/or ARB/ACEI and/or statin (in the maximum tolerated doses that ranged between 20 and 80 mg)	

DM—diabetes mellitus; T2DM—type 2 diabetes mellitus; SGLT-2i—sodium glucose loop transporter 2 inhibitor; GLP-1 RA—glucagon-like peptide 1 receptor agonist; BB—beta-blockers; CCB—calcium-channel blockers; ARB—angiotensin receptor blockers; and ACEI—angiotensin-converting enzyme inhibitors.

**Table 2:** Data about the patient’s distribution, demography, metabolic control, and cardiovascular disease incidence and treatment.

	Metformin	SGLT-2i	GLP1-RA
No of patients (% of total)	167 (41.2%)	119 (29.4%)	119 (29.4%)
Insulin treatment (%)	16 (9.58%)	15 (12.6%)	61 (51.26%)
Female (%)	65 (38.9%)	34 (28.57%)	57 (47.9%)
Mean age (years)	57.9 ± 10.1	59.2 ± 9.09	59.2 ± 9.09
Urban settlement (%)	113 (67.66%)	98 (82.35%)	88 (73.95%)
Active smoker (%)	29 (17.36%)	27 (22.68%)	27 (22.68%)
HbA1c (%)	7.4 ± 1.2	7.4 ± 1.4	7.4 ± 1.4
Body weight (kg)	94.2 ± 19.4	92.5 ± 16.6	92.5 ± 16.6
BB	94 (56.28%)	78 (65.54%)	74 (62.18%)
CCB	43 (25.74%)	28 (23.52%)	28 (23.52%)
ACEI/ARB	104 (62.27%)	85 (71.42%)	94 (78.98%)
Statin	135 (80.83%)	104 (87.39%)	106 (89%)
Atherosclerotic cardiovascular disease (%)	46 (27.54%)	51 (42.85%)	42 (35.29%)
Myocardial infarction history (%)	8 (4.79%)	6 (5.04%)	4 (3.36%)

SGLT-2i—sodium glucose loop transporter 2 inhibitor; GLP-1 RA—glucagon-like peptide 1 receptor agonist; BB—beta-blockers; CCB—alcium channel blockers; ACEI—angiotensin-converting enzyme inhibitors; and ARB—angiotensin receptor blockers.

**Table 3:** Baseline characteristics of safety.

	Metformin	SGLT-2i	GLP1-RA
Palpitations	0	2 (1.68%)	2 (1.68%)
Hypoglycemia	0	1 (0.84%)	1 (0.84%)
Gastrointestinal AR—nausea, diarrhea, vomiting, pancreatitis	0	0	0
Urinary tract infections	0	0	0
Creatinine (mg/dL)	0.75 ± 0.2	0.77 ± 0.19	0.77 ± 0.19
eGFR (mL/min/1.73 m^2^)	97.8 ± 16.4	96.3 ± 15.9	96.4 ± 15.9
Urea (mg/dL)	34.4 ± 11.45	34.81 (17, 89)	34.81 (17, 89)
UACR (mg/g creatinine)	72.6 (1, 4899)	31.3 (1, 648)	28.9 (1, 648)
GOT (UI/L)	24 (11, 184)	20 (7, 75)	20 (7, 75)
GPT (UI/L)	30 (4, 154)	26 (7, 120)	26 (7, 120)

SGLT-2i—sodium glucose loop transporter 2 inhibitor; GLP-1 RA—glucagon-like peptide 1 receptor agonist; AR—adverse reaction; eGFR—estimated glomerular filtration rate; UACR—urinary albumin to creatinine ratio; GOT—aspartate amino-transferase; and GPT—alanine aminotransferase.

**Table 4:** 6-month characteristics of safety.

	Metformin	SGLT-2i	GLP1-RA
Palpitations	0	0	0
Hypoglycemia	0	1 (0.84%)	1 (0.84%)
Gastrointestinal AR–nausea, diarrhea, vomiting, pancreatitis	0	0	5 (4.2%)
Urinary tract infections	0	2 (1.68%)	0
Creatinine (mg/dL)	0.78 ± 0.21	0.76 ± 0.17	0.75 ± 0.16
eGFR (mL/min/1.73 m2)	95.4 ± 17.9	95.7 ± 15.7	95.88 ± 15.6
Urea (mg/dL)	35.9 ± 11.22	32.82 (17, 65)	32.82 (17, 65)
UACR (mg/g creatinine)	62.5 (1, 4800)	35.7 (1, 944)	31.8 (1, 944)
GOT (UI/L)	21 (9, 88)	26 (10, 415)	26 (10, 415)
GPT (UI/L)	26 (6, 119)	28 (8,197)	28 (8, 197)

SGLT-2i—sodium glucose loop transporter 2 inhibitor; GLP-1 RA—glucagon-like peptide 1 receptor agonist; AR—adverse reaction; eGFR—estimated glomerular filtration rate; UACR—urinary albumin to creatinine ratio; GOT—aspartate amino-transferase; and GPT—alanine aminotransferase.

**Table 5:** 12-month characteristics of safety.

	Metformin	SGLT-2i	GLP1-RA
Palpitations	0	0	0
Hypoglycemia	0	0	0
Gastrointestinal AR–nausea, diarrhea, vomiting, pancreatitis	0	0	8 (6.72%)
Urinary tract infections	0	3 (2.52%)	0
Creatinine (mg/dL)	0.79 ± 0.22	0.75 ± 0.17	0.75 ± 0.17
eGFR (mL/min/1.73 m^2^)	94.7 ± 18.7	96.2 ± 17.1	96.3 ± 17.1
Urea (mg/dL)	37.03 ± 12.83	33.91 (17, 65)	34 (17, 65)
UACR (mg/g creatinine)	58.6 (1, 3320)	42.1 (1, 1068)	37.8 (1, 1068)
GOT (UI/L)	22 (10, 90)	21 (10, 143)	21 (10, 143)
GPT (UI/L)	27 (6, 147)	24 (8, 116)	24 (8,116)

SGLT-2i—sodium glucose loop transporter 2 inhibitor; GLP-1 RA—glucagon-like peptide 1 receptor agonist; AR—adverse reaction; eGFR—estimated glomerular filtration rate; UACR—urinary albumin to creatinine ratio; GOT—aspartate amino-transferase; and GPT—alanine aminotransferase.

**Table 6:** Safety parameters within metformin, SGLT-2i, and GLP-1 Ra groups at V6M and V12M as compared to V0M, and V12M as compared to V6M.

	V6M Compared to V0M			V12M Compared to V0M			V12M Compared to V6M		
	Metformin	SGLT-2i	GLP-1 RA	Metformin	SGLT-2i	GLP-1 RA	Metformin	SGLT-2i	GLP-1 RA
Creatinine (mg/dL)	*p* = 0.011	NS	NS	*p* < 0.001	NS	NS	NS	NS	NS
Adjustment for age *	Β = 0.007 (R^2^ = 12.4%), *p* < 0.001)	NS	NS	Β = 0.304 (R^2^ = 9.4%), *p* < 0.001)	NS	NS	Β = 0.318 (R^2^ = 10.1%), *p* < 0.001)	NS	NS
eGFR (mL/min/1.73 m^2^)	*p* = 0.003	NS	NS	*p* < 0.001	NS	NS	NS	NS	NS
Adjustment for age *	Β = −0.694 (R^2^ = 48.4%), *p* < 0.001)	NS	NS	Β = −0.622 (R^2^ = 38.6%), *p* < 0.001)	NS	NS	Β = −0.622 (R^2^ = 38.7%), *p* < 0.001)	NS	Β = 0.197 (R^2^ = 3.9%), *p* = 0.049)
Urea (mg/dL)	NS	*p* = 0.007	*p* = 0.007	*p* < 0.001	NS	NS	*p* = 0.046	NS	NS
Adjustment for age *	Β = 0.445 (R^2^ = 19.8%), *p* < 0.001)	NS	NS	Β = 0.478 (R^2^ = 23.4%), *p* < 0.001)	NS	NS	Β = 0.445 (R^2^ = 19.8%), *p* < 0.001)	NS	NS
GOT (UI/L)	*p* = 0.028	NS	NS	NS	NS	NS	NS	NS	NS
Adjustment for age *	NS	Β = −0.255 (R^2^ = 5.3%), *p* = 0.014)	Β = −0.229 (R^2^ = 5.4%), *p* = 0.013)	NS	NS	NS	NS	NS	NS
GPT (UI/L)	*p* = 0.037	NS	NS	NS	NS	NS	NS	NS	NS
Adjustment for age *	NS	Β = −0.378 (R^2^ = 5.4%), *p* = 0.015)	Β = −0.224 (R^2^ = 5.4%), *p* = 0.015)	Β = −0.232 (R^2^ = 5.4%), *p* = 0.005)	NS	NS	NS	NS	NS

* Age, gender and comedication were assessed in the model, but only for age was met a *p* < 0.05; NS = not significant, *p* < 0.05; SGLT-2i—sodium glucose loop transporter 2 inhibitor; GLP-1 RA—glucagon-like peptide 1 receptor agonist; eGFR—estimated glomerular filtration rate, UACR—urinary albumin to creatinine ratio; V0M—initial visit; V6M—6 months visit; V12M—12 months visit; GOT—aspartate amino-transferase; GPT—alanine aminotransferase.

**Table 7:** Safety parameters between metformin and SGLT-2i and between metformin and GLP-1 RA groups at V6M and V12M, as compared to V0M, and at V12M as compared to V6M.

	SGLT-2i Group Compared to Metformin Group	Adjustment for Age *	GLP-1 RA Group Compared to Metformin Group	Adjustment for Age *
V0M				
Creatinine (mg/dL)	NS	NS	NS	NS
eGFR (mL/min/1.73 m^2^)	NS	β = −0.501 (R^2^ = 25.4%), *p* < 0.001	NS	β = −0.518 (R^2^ = 25.9%), *p* < 0.001
Urea (mg/dL)	NS	β = 0.405 (R^2^=16.5%), *p* < 0.001	NS	β = −0.403 (R^2^ = 16.4%), *p* < 0.001
UACR (mg/g creatinine)	NS	NS	NS	NS
GOT (UI/L)	NS	NS	NS	β = −0.204 (R^2^ = 8.3%), *p* = 0.027
GPT (UI/L)	NS	β = −0.28 (R^2^ = 4.4%), *p* = 0.022	NS	β = −0.253 (R^2^ = 8.1%), *p* = 0.007
V6M				
Creatinine (mg/dL)	NS	NS	NS	NS
eGFR (mL/min/1.73 m^2^)	NS	β = −0.424 (R^2^ = 18.1%), *p* < 0.001	NS	β = −0.446 (R^2^ = 19.1%), *p* < 0.001
Urea (mg/dL)	*p* = 0.02	β = 0.304 (R^2^ = 11.6%), *p* = 0.001	*p* = 0.02	β = 0.3 (R^2^ = 0.2%), *p* = 0.002
UACR (mg/g creatinine)	NS	NS	NS	NS
GOT (UI/L)	NS	NS	NS	NS
GPT (UI/L)	NS	NS	NS	NS
V12M				
Creatinine (mg/dL)	NS	β = 0.209 (R^2^ = 6.5%), *p* = 0.035	NS	NS
eGFR (mL/min/1.73 m^2^)	NS	β = −0.587 (R^2^ = 34.5%), *p* < 0.001	NS	NS
Urea (mg/dL)	NS	β = 0.492 (R^2^ = 24.7%), *p* < 0.001	NS	NS
UACR (mg/g creatinine)	NS	NS	NS	NS
GOT (UI/L)	NS	NS	NS	NS
GPT (UI/L)	NS	NS	NS	NS

* Age, gender, and comedication were assessed in the model, but only for age was met with a *p* < 0.05; NS = not significant, *p* < 0.05; SGLT-2i—sodium glucose loop transporter 2 inhibitor; GLP-1 RA—glucagon-like peptide 1 receptor agonist; V0M—baseline visit; V6M—6 months visit; V12M—12 months visit; eGFR—estimated glomerular filtration rate; UACR—urinary albumin to creatinine ratio; GOT—aspartate amino-transferase; and GPT—alanine aminotransferase.

## Data Availability

Not applicable.
